# Good Syndrome, Bad Problem

**DOI:** 10.3389/fonc.2014.00307

**Published:** 2014-11-10

**Authors:** Bianca Martinez, Sarah K. Browne

**Affiliations:** ^1^Immunopathogenesis Section, Laboratory of Clinical Infectious Diseases, National Institute of Allergy and Infectious Diseases, National Institutes of Health, Bethesda, MD, USA

**Keywords:** thymoma, adult-onset immunodeficiency, thymic neoplasia, anti-cytokine autoantibodies, opportunistic infection

Good syndrome (GS), which is classically defined as the triad of thymoma, immunodeficiency, and hypogammaglobulinemia, was first characterized by Robert Alan Good, a pioneer in the field of immunodeficiency diseases, who recognized the crucial role that the thymus plays in the development of the immune system (1). The definition of this entity remains vague, in part because of the protean immunological manifestations of thymic epithelial neoplasms. How the thymus influences the precise balance of immune reactivity that is so critical to both host-defense and self-tolerance remains to be fully understood. Accordingly, immunologic manifestations because of thymic epithelial tumors can range from nothing to severe autoimmunity, immunodeficiency, or both, the spectrum of which is broad and patient-specific (Table 1). Further, patients may have opportunistic infection without gross immunologic lab abnormalities (2), and conversely, may have relatively few problems, despite an abnormal immune profile (3). Nevertheless, we can look to the immunology and infectious complications in individual patients, to identify where their immune defects are most concentrated and how to go about treating them.

**Table 1:** **Immunologic abnormalities reported in thymoma**.

Laboratory features	Abnormality/activity	Clinical associations	Management	Comments
**LYMPHOCYTE SUBSETS**				
CD20^+^ B cells	Decreased in peripheral blood (2, 3)	Immunodeficiency	Consider vaccine challenge to evaluate ability to mount antibody response	May be independent of hypogammaglobulinemia
CD20^+^/CD27^+^ memory B cells	Decreased in peripheral blood (2)	Unknown, often in setting of total B cell lymphopenia	As above	As above
CD4^+^ T cells	May be increased or decreased in peripheral blood (2, 3)	Elevated levels associated with autoimmunity	Therapy targeting clinical manifestations	CMV encephalitis reported in the presence of normal CD4^+^ T cell counts (18)
CD8^+^ T cells	May be increased or decreased in peripheral blood (2, 3)	Elevated levels associated with immunodeficiency	Therapy directed at clinical manifestations; consider secondary prophylaxis if history of opportunistic infection	The accumulation of CD8^+^CD45RA^+^ T cells can be used to monitor clinical stages of immunodeficiency in thymoma (19)
CD16^+^ or CD56^+^ NK cells	May be increased or decreased in peripheral blood (2, 3)	Unknown	No specific therapy indicated	Low or dysfunctional NK cells associated with herpes virus infection
**IMMUNOGLOBULINS (Ig)**				
IgG	May be increased or decreased in peripheral blood (2, 3)	Recurrent bacterial sinopulmonary infections	If there is an inadequate antibody response, or history of severe or recurrent infection, immunoglobulin replacement therapy may be started (18)	Even if immunoglobulins normal or high, consider vaccine challenge to evaluate ability to mount antibody response, particularly if history of severe or recurrent infections (18)
IgA	May be increased or decreased in peripheral blood (2, 3)	Unknown	No specific therapy indicated	None
IgM	May be increased or decreased in peripheral blood (2, 3)	Unknown	No specific therapy indicated	None
**ANTI-CYTOKINE AUTOANTIBODIES**				
Anti-IFNα	Prevents IFNα-induced pSTAT-1 and pSTAT-4 *in vitro* (2)	Unknown	IFNα given to one patient with disseminated zoster	Associated with one case of disseminated *severe varicella zoster* infection in patient without thymoma; thymoma patients may have disseminated or localized varicella reactivation
Anti-IFNβ	Prevents IFNβ-induced pSTAT-1 *in vitro* (2)	Unknown	No specific therapy indicated	One case of activating anti-IFNβ autoantibodies (unpublished data)
Anti-IFNω	Prevents IFNω-induced pSTAT-1 (unpublished data)	Unknown	No specific therapy indicated	Autoantibodies against type I IFNs common in APECED suggesting etiologic role of gene *AIRE* for origin of some anti-cytokine autoantibodies
Anti-IL-1α	Prevents PHA-induced IFNγ production by T cells (2)	Unknown	No specific therapy indicated	Can be seen in normal hosts (12)
Anti-IL-12p70	Prevents IL-12-induced pSTAT-4 *in vitro*; prevented IL-12-induced IFNγ *in vitro* (2)	Unknown	Targeted anti-infectives	Associated with one case of disseminated *Burkholderia gladioli* infection in patient without thymoma
Anti-12p35	Same as effects seen with anti-IL-12p70 autoantibodies	Unknown	No specific therapy indicated	None
Anti-IL-12p40	Same as effects seen with anti-IL-12p70	Unknown	No specific therapy indicated	The p40 subunit is common to IL-23, raising possibility for activity beyond neutralization of IL-12
IL-17A or anti-IL-17F	Prevents IL-17-induced IL-6 production in HFF cells (2)	CMC (14)	Topical or systemic antifungals	Associated with CMC in APECED syndrome (14); may become treatment-refractory
IL-22	Not assessed	CMC (14)	Topical or systemic antifungals	Associated with CMC in APECED syndrome; IL-22^+^/IL-17^−^cells protect epithelial surfaces and show skin-homing properties, which may explain the mucocutaneous focus of the candidiasis (14)
TNFα	Not assessed (2)	Unknown	Unknown	Unknown

*NK, natural killer; IFN, interferon; APECED, autoimmune polyendocrinopathy candidiasis ectodermal dystrophy; *AIRE*, autoimmune regulator; IL, interleukin; CMC, chronic mucocutaneous candidiasis; TNFα, tumor necrosis factor alpha*.

The critical role that the thymus plays in T cell education likely explains the observation of coincident autoimmunity and immunodeficiency in thymoma, reflecting T cells that are both over-reactive to self and under-responsive to pathogens. The importance of the T cell in directing B cell responses is also apparent in the immunopathology of thymoma. Consistent with T cell immunodeficiency, patients with thymoma can develop pneumocystis pneumonia, cytomegalovirus, mucocutaneous candidiasis, varicella zoster reactivation (both localized and systemic), Kaposi’s sarcoma, and progressive multifocal leukoencephalopathy, cryptococcosis, and non-tuberculous mycobacteria (2, 4, 5). Underscoring the B cell component of this disease, the cardinal manifestation of GS is hypogammaglobulinemia. In fact, the most prominent clinical characteristics of GS includes an increased susceptibility to sinopulmonary bacterial infections with encapsulated organisms (*Haemophilus influenza* and *Streptococcus pneumonia*), which is clearly associated with hypogammaglobulinemia. Even outside classical GS, a tendency toward B cell dysfunction is apparent in the observation that anti-acetylcholine receptor autoantibody-associated myasthenia gravis is the most frequent autoimmune complication of thymoma (6). B cell lymphopenia is also common and in some cases has even led to the initial diagnosis of thymoma (7, Allergy and Asthma Proceedings). Together, these observations underscore the complex interrelatedness of T and B lymphocyte biology.

Another potential mechanism of infection susceptibility is presence of anti-cytokine autoantibodies (2, 8–10). It is clear that anti-cytokine autoantibodies are an important and emerging mechanisms of adult-onset immunodeficiency (11) and can be responsible for severe opportunistic infection in previously healthy adults (12, 13). Interestingly, mucocutaneous candidiasis has been seen in association with anti-IL-17 and anti-IL-22 autoantibodies in autoimmune polyendocrinopathy candidiasis ectodermal dystrophy (APECED) syndrome (14, 15), caused by a Mendelian deficiency of the autoimmune regulator (AIRE) gene. AIRE is critical for the negative selection of autoreactive T cells in the thymus (16) and likely explains much of the profound and diverse autoimmune phenomena that are typical of APECED. This compelling link between autoimmunity and immunodeficiency may also be relevant to thymoma where defective AIRE expression has been recognized (17), as has a predisposition to mucocutaneous candidiasis in association with anti-IL-17 and IL-22 autoantibodies (2, 14).

Immunological evaluation can include an assessment of quantitative immunoglobulins, as well as B and T lymphocyte subsets. If the clinical presentation points to antibody deficiency, a vaccine challenge may help evaluate the patient’s ability to generate an appropriate humoral response, which can be impaired even with relatively normal IgG levels. Testing for anti-cytokine autoantibodies is done in specialized laboratories on a research basis.

Treatment of infection generally focuses on targeted antimicrobial therapy. In the case of recurrent sinopulmonary infections associated with hypogammaglobulinemia, intravenous immunoglobulin (IVIg) can be an effective prophylactic measure. IVIg has also been used to augment the immune response in the case of CMV infection (5). It can also be effective in treating anti-acetylcholine receptor-associated myasthenia gravis, although the mechanistic explanation for this benefit remains elusive. Secondary prophylaxis could be considered in patients under certain circumstances such as those who have had pneumocystis pneumonia.

A better understanding of the underlying immunological defects in thymoma will enhance therapeutic options, such as primary prophylaxis or immunomodulation. Furthermore, despite the focality of their primary immunologic lesion (the neoplastic thymic epithelial cells), patients with thymoma can demonstrate fascinating clinical overlaps with many other diseases, from rheumatologic, hematologic, and pulmonary disorders, to primary and congenital immunodeficiency, and far beyond. Thus, understanding thymoma, as Dr. Good so astutely recognized in decades past, will provide an important opportunity that should not be missed, to improve care of these complex patients, and to shed light on fundamental principals of human immunology.

## Conflict of Interest Statement

The authors declare that the research was conducted in the absence of any commercial or financial relationships that could be construed as a potential conflict of interest.
